# De novo design of monomeric helical bundles for pH‐controlled membrane lysis

**DOI:** 10.1002/pro.4769

**Published:** 2023-10-16

**Authors:** Nicolas Goldbach, Issa Benna, Basile I. M. Wicky, Jacob T. Croft, Lauren Carter, Asim K. Bera, Hannah Nguyen, Alex Kang, Banumathi Sankaran, Erin C. Yang, Kelly K. Lee, David Baker

**Affiliations:** ^1^ Institute for Protein Design University of Washington Seattle Washington USA; ^2^ Molecular Life Sciences Technical University of Munich Munich Germany; ^3^ Department of Bioengineering University of Washington Seattle Washington USA; ^4^ Department of Biochemistry University of Washington Seattle Washington USA; ^5^ Department of Medicinal Chemistry University of Washington Seattle Washington USA; ^6^ Molecular Biophysics and Integrated Bioimaging Lawrence Berkeley National Laboratory Berkeley California USA; ^7^ Biological Physics, Structure and Design Graduate Program University of Washington Seattle Washington USA; ^8^ Department of Microbiology University of Washington Seattle Washington USA; ^9^ Howard Hughes Medical Institute University of Washington Seattle Washington USA

**Keywords:** coiled‐coil, endosomal escape, membrane disruption, pH responsive, protein design

## Abstract

Targeted intracellular delivery via receptor‐mediated endocytosis requires the delivered cargo to escape the endosome to prevent lysosomal degradation. This can in principle be achieved by membrane lysis tightly restricted to endosomal membranes upon internalization to avoid general membrane insertion and lysis. Here, we describe the design of small monomeric proteins with buried histidine containing pH‐responsive hydrogen bond networks and membrane permeating amphipathic helices. Of the 30 designs that were experimentally tested, all expressed in *Escherichia coli*, 13 were monomeric with the expected secondary structure, and 4 designs disrupted artificial liposomes in a pH‐dependent manner. Mutational analysis showed that the buried histidine hydrogen bond networks mediate pH‐responsiveness and control lysis of model membranes within a very narrow range of pH (6.0–5.5) with almost no lysis occurring at neutral pH. These tightly controlled lytic monomers could help mediate endosomal escape in designed targeted delivery platforms.

## 1 INTRODUCTION

The ability to escape the endosome is a critical property of successful targeted delivery platforms. Adenoviruses utilize the decrease in pH upon endosomal acidification as an environmental trigger to expose membrane disrupting sequence motifs, enabling viral capsids to escape the endosome and enter the cytosol (Maier et al., 2010; Staring et al., 2018; Wiethoff et al., 2005). Many engineering efforts have used peptides to permeate membranes by various mechanisms and increase cytosolic delivery of cargo (Akishiba et al., 2017; Chen et al., 2021; Rhys et al., 2022; Sakamoto et al., 2021). Membrane lysis has been achieved by peptides forming pores in acidic environments (Wiedman et al., 2015; Zhang et al., 2015), but fusion of free peptides to cargo can be complicated, and the size of the cargo is limited by the pore size. Other membranolytic peptides preferentially lyse the endosomal membrane on the basis of their negative charge (Akishiba et al., 2017). While co‐incubation of these peptides and cargo works for in vitro models, systemic administration would lead to untargeted uptake into any cell type and cause uncontrolled endosome damage.

The de novo designed homotrimer pH‐responsive bundles‐2 (pRO‐2) I56V with histidine‐containing hydrogen bond networks (nine histidines in total) was previously found to disrupt liposome membranes in a pH‐dependent manner and liposome lysis was impaired by mutations in the C‐terminal amphipathic helix (Boyken et al., 2019). However, hydrophobic and hydrophilic residues in the C‐terminal helix were not spatially separated resulting in a low helical hydrophobic moment (Eisenberg et al., 1982) (Figure 1a). We reasoned that a greater spatial separation of hydrophobic and hydrophilic residues could result in helices with increased amphiphilicity and consequently more efficient membrane disruption (Figure 1a). The homo‐trimeric nature of pRO‐2 I56V also limits its use‐case for incorporation in asymmetric scaffolds. Here, we aimed to create small monomeric proteins that reveal membrane disrupting motifs in acidic environments such as the late endosome. These proteins could improve the endosomal escape of cell‐specific targeting assemblies such as nanocages displaying receptor‐targeting domains.

**FIGURE 1:**
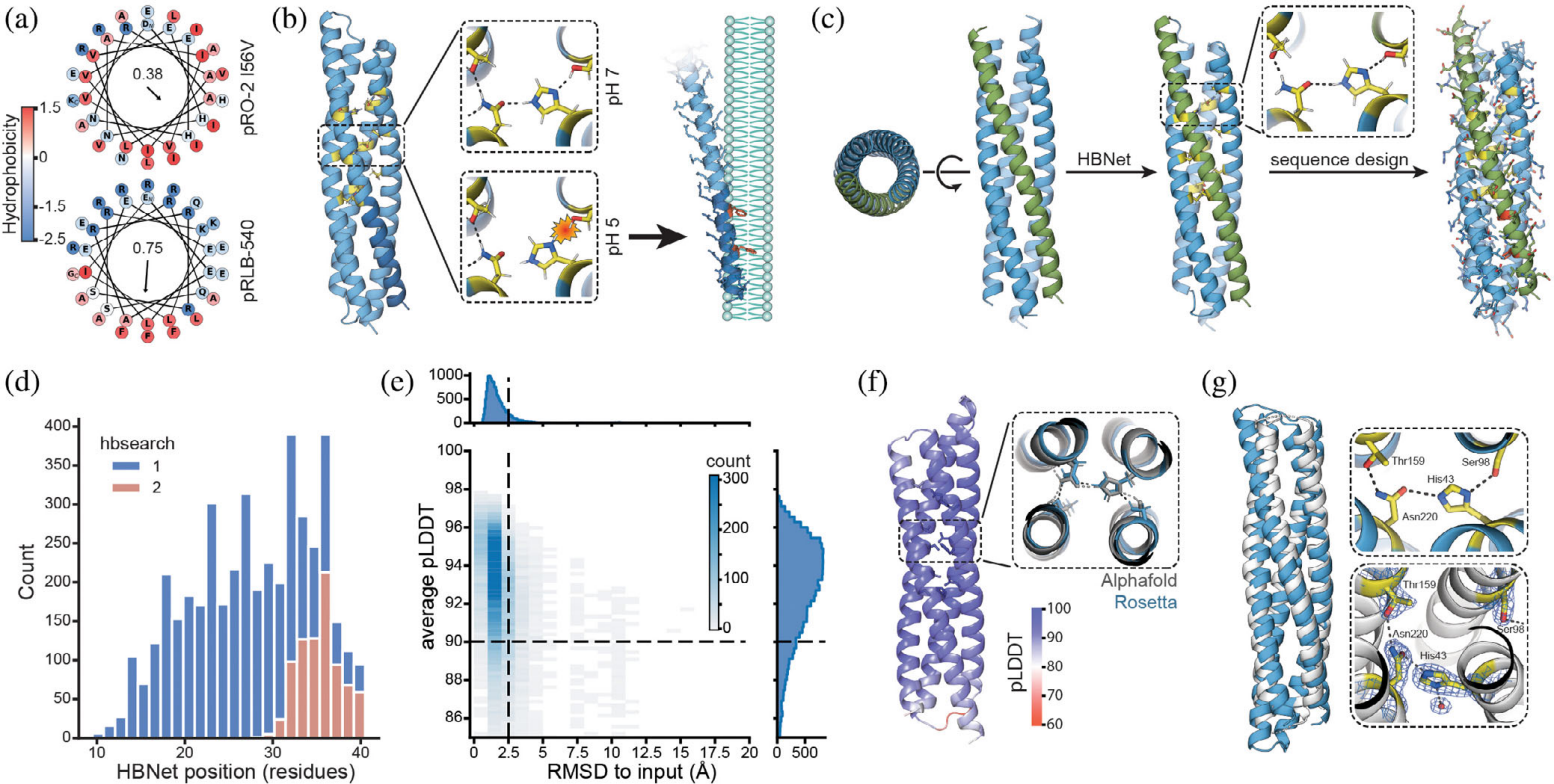
Design strategy and computational results. (a) Wheel projections of the C‐terminal helices from pH‐responsive bundles‐2 (pRO‐2) I56V (Boyken et al., 2019) (top, previous work) and pH‐responsive lytic bundles (pRLB)‐540 (bottom, this work) with amino acids colored by their hydrophobicity (Eisenberg et al., 1984). Hydrophobic moment is shown in the center of the respective wheel. Arrow represents the magnitude and direction of the hydrophobic moment. (b) Protonation of the buried histidine residue in the pH‐responsive hydrogen bond networks (yellow) separates the individual helices from each other and exposes amphipathic helices. The C‐terminal region (dark blue) carries phenylalanines (red), which increase membrane interaction. Exposed amphipathic helices insert into phospho bilayer membranes, inducing curvature or solubilizing membrane particles in a detergent‐like manner. (c) Design strategy: starting from a library of parametrically defined four helix bundles, pH‐responsive hydrogen bond networks (yellow) were positioned in the core of the bundle using the HBNet mover. Individual helices of bundles containing two to three pH‐responsive hydrogen bond networks were looped and the remaining sequence was designed while conserving the network residues. Phenylalanines (red) were enriched in the C‐terminal helix (green). (d) Position of the hydrogen bond networks. Distance is measured in residues from the N‐terminus to the first residue participating in the hydrogen bond network. Searching for buried hydrogen bond networks over the entire length of the bundle (1) and only the top part, opposite to N‐ and C‐terminus of the bundle (2). (e) Designed structures are confidently‐predicted by AlphaFold2 (AF2) and are in close agreement with the Rosetta design model (root‐mean‐square deviation [RMSD] to input). Dashed lines indicate cutoffs used for selecting designs for experimental testing (nota bene outliers not shown). (f) AF2 predictions showed structures with the hydrogen bond networks matching the design models, and with high confidence in rotameric positions of residues participating in the hydrogen bond networks. (g) Crystal structure (Protein Data Bank 8GL3, white) and design model (blue). The zoom in views show design model (top, blue) and experimentally determined hydrogen bond networks (bottom, white). Electron density is shown as a blue mesh for the experimentally determined structure (bottom).

## 2 RESULTS

Protonation of buried histidine residues participating in hydrogen bond networks should be structurally disruptive, and, as with our previously designed trimeric helical bundles, we hypothesized that it could be used as a pH controlled mechanism for the exposure of caged amphipathic helices for membrane lysis (Figure 1b). We used parametric backbone generation to produce scaffolds on which to base such pH‐responsive lytic bundles (pRLBs). Four helix bundle topologies were generated by sampling parameters of the Crick‐generating equations (Crick, 1953; Grigoryan & Degrado, 2011; Huang et al., 2014) in the range of those of native proteins. Next, we installed buried hydrogen bond networks containing histidine residues using the Rosetta HBNet mover (Boyken et al., 2016, 2019; Maguire et al., 2018) (Figure 1c; protonation of buried histidine residues in hydrogen bond networks was shown previously to mediate dissociation of homo‐oligomers [Boyken et al., 2019]). Two or three pH‐responsive hydrogen bond networks were designed in the core of the bundle and the remaining sequence of the bundle was designed using Rosetta (Alford et al., 2017; Bhardwaj et al., 2016; Dang et al., 2017; Fleishman et al., 2011; Hosseinzadeh et al., 2017; Khatib et al., 2011; Maguire et al., 2021; Tyka et al., 2011) and ProteinMPNN (Dauparas et al., 2022) while keeping the residues participating in the hydrogen bond networks fixed.

In a first design round, we distributed pH‐responsive hydrogen bond networks over the entire length of the bundle (Figure 1d; hbsearch 1). In a second round of design, we restricted the hydrogen bond networks to a limited section opposite to the C‐ and N‐terminus (top region in illustrations) of the bundle, thus spatially separating polar residues of the hydrogen bond networks from the amphipathic motif (Figure 1d; hbsearch 2). We hypothesized that in an acidic environment like the late endosome, protonation of the histidine residues participating in the hydrogen bond networks would compromise hydrophobic packing of the bundle and cause the helices of the bundle to dissociate. Upon unfolding of the tertiary structure, the hydrophobic faces of the amphipathic helices would become exposed, allowing interaction of hydrophobic residues with membranes and eventually membrane lysis. Phenylalanines were placed in the C‐terminal amphipathic region of the bundle opposite to the hydrogen bond networks (Figure 1c) to increase association of the amphipathic helices with membranes and membrane disruption potential (Chen et al., 2009; Rosa et al., 2015; Shahmiri et al., 2017). We predicted structures of the Rosetta designed sequences with AlphaFold2 (AF2) (Jumper et al., 2021) using speedups from ColabFold (Mirdita et al., 2022). The predicted AF2 structures all had overall high predicted local distance difference test (pLDDT) scores (averaged per residue pLDDT over all residues, mean = 92.5) and low Cα root‐mean‐square deviation (RMSD) relative to the Rosetta design model (mean = 1.8 Å; Figure 1e). Side chains participating in the hydrogen bond networks were predicted with high confidence (pLDDT >95) and low RMSD (<1.5 Å) to the Rosetta design model (Figure 1f). As AF2 can predict the correct side chain rotamer for high confidence backbones (as assessed by local pLDDT) (Jumper et al., 2021), we filtered the designs on metrics calculated on the side chain predictions. We selected 30 designs for experimental characterization based on the number of hydrogen bonds between residues participating in the designed hydrogen bond networks, the number of hydrogen bonds specifically involving the histidine residues, the number of buried hydrogen bond donors/acceptors not satisfied by hydrogen bonds (Adolf‐Bryfogle et al., 2021) and the energy of hydrogen bonds in the networks.

### 2.1 Characterization of designed proteins

Synthetic genes encoding 30 designs (Table S1) were expressed in *Escherichia coli*, purified by nickel‐nitrilotriacetic acid (Ni‐NTA) affinity chromatography followed by size exclusion chromatography (SEC). All 30 designs expressed and were soluble, and 13 of the 30 purified designs were monomeric, as determined by SEC coupled to multi‐angle light scattering (SEC‐MALS) (Figures 2 and S1). The monomeric designs had circular dichroism (CD) spectra at pH 7.4 consistent with their secondary structures (Figure 2). Secondary structures of pRLB‐540 and pRLB‐539 were found to be thermostable as assessed by the retention of their secondary structure up to 95°C (Figure S2). Hydrodynamic diameters of the monomeric proteins were determined by dynamic light scattering (DLS) and were distributed around the expected diameter of the respective monomer at pH 7.2. In contrast, the distribution clearly shifted to aggregated particles at pH 5, while the secondary structure as estimated by CD was not affected by acidic conditions, suggesting that acidification affects the structure of the bundles on a tertiary level while maintaining helical secondary structure propensity (Figure 2). We solved the crystal structure of design 519 to a resolution of 2.3 Å (PDB 8GL3; Figure 1g). The backbone of the design model was in close agreement with the experimentally determined structure with an overall Ca RMSD of 2.7 Å. However, the hydrogen bond network around the buried histidine residue differs from the design model; the δ1 nitrogen of the histidine 43 forms a hydrogen bond to a water molecule instead of the serine 98 residue (AF2 predicted the hydrogen bond present in the design model of pRLB‐519, not the crystal structure, perhaps because it does not explicitly capture water molecules). Consistent with the alteration in the hydrogen bond network, pRLB‐519 had low pH‐responsiveness (Figure S1), and hence was not characterized further.

**FIGURE 2:**
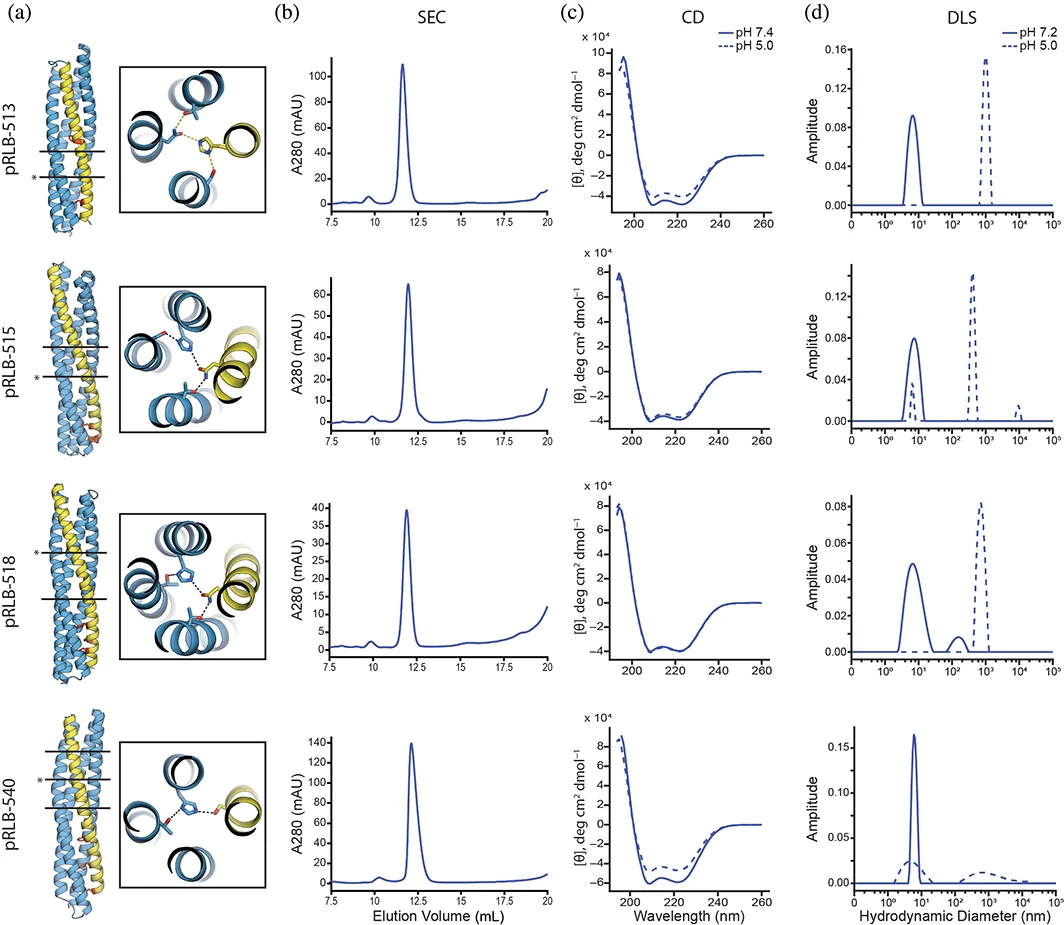
Experimental characterization of designed proteins. (a) Model of the four designs 513, 515, 518, and 540 with zoom in views showing the indicated (*) pH‐responsive hydrogen bond network. Black lines represent hydrogen bond network layers. The C‐terminal helix (yellow) carries phenylalanines for membrane disruption (red). Size exclusion chromatography chromatogram (A280 = absorbance at 280 nm, AU = arbitrary units) (b), circular dichroism (CD) spectra (c), and dynamic light scattering (DLS) results (d) of the four proteins are shown. CD spectra and DLS data were collected at close to neutral pH and pH 5 (see Section 4 for details).

### 2.2 Designs disrupt liposomes in a pH‐dependent manner

The capacity of the designed bundles to disrupt membranes was assessed with artificial liposomes serving as model membranes. Purified proteins at 4 μM were incubated with synthetic liposomes encapsulating sulforhodamine B (SRB) at self‐quenching concentrations over a range of pH values. Disruption of liposomes was determined by measuring fluorescence of SRB upon dye dequenching upon release from the liposomes (SRB fluorescence is not affected by pH values used in this experiment) (Gui & Lee, 2018).

Four designs were found to disrupt liposomes at pH 5 (Figure 3a). At pH 5, designs pRLB‐518 and pRLB‐540 caused 50% and 60% liposome lysis respectively, and designs pRLB‐513 and 515 led to 40% liposome lysis. Liposome lysis was found to be stringently pH‐dependent for pRLB‐540, with almost no lysis occurring at pH 7.3 (Figure 3b). Comparison of protein pRLB‐540 to the most active of our previously designed pH‐dependent trimers with similar mechanisms revealed comparable levels of liposome lysis at pH 5 and 5.5, but a more tightly pH‐controlled lytic activity for pRLB‐540 at close to neutral pH (see Figure S3). Mutating the three histidine residues in pLRB‐540 to asparagine impaired liposome disruption at low pH, consistent with the design concept (pLRB‐540‐noHis; Figure 3a).

**FIGURE 3:**
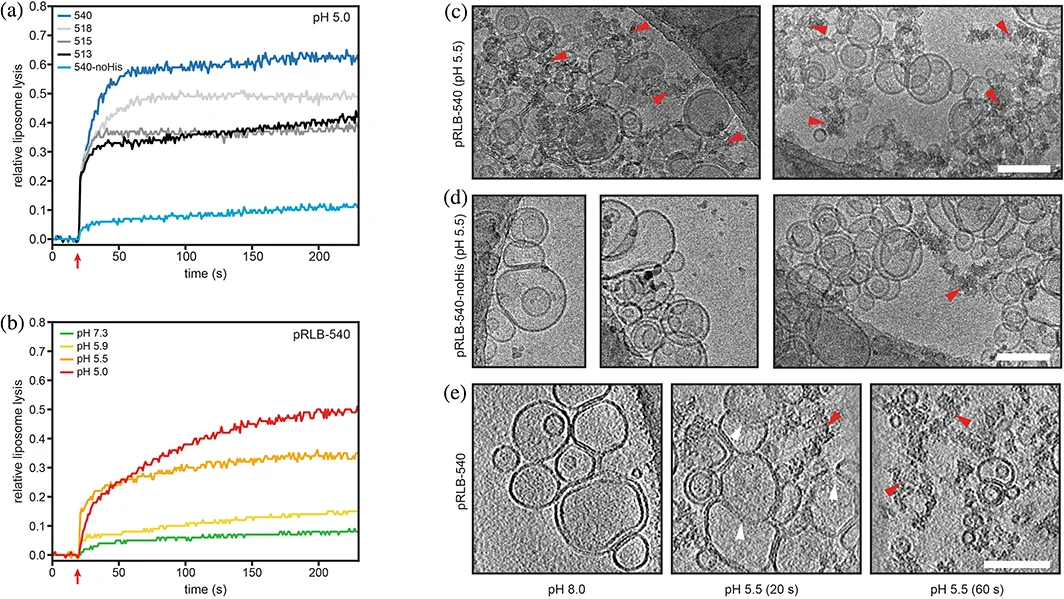
Liposome disruption by pH‐responsive lytic bundles (pRLBs). (a and b) Proteins were incubated with synthetic liposomes encapsulating sulforhodamine B (SRB) at self‐quenching concentrations; liposome disruption was investigated by measurement of SRB fluorescence. Red arrows indicate acidification to the respective pH. (a) All four designs tested were able to lyse liposomes at pH 5 to different extent and plateaued after 60 s. Mutation of the histidine residue in the pH‐responsive hydrogen bond network to asparagine impairs lytic activity. (b) Liposome lysis of pRLB‐540 is tightly controlled by pH. Cryo‐electron microscopy was used to visualize liposomes mixed with pRLB‐540 (c) and pRLB‐540‐noHis (d). Representative images of the respective condition are shown. The mixture was brought to pH 5.5, and plunge‐frozen after a 60 s incubation, and imaged by conventional cryo transmission electron cryomicroscopy. Cryo‐electron tomography was used to image pRLB‐540 mixed with liposomes at pH 8.0, and after 20 and 60 s incubation at pH 5.5 (e). Images shown in panel (e) are averages of 17 Å thick slices in the *Z*‐plane. White arrowheads indicate liposome membranes in an intermediate state of disruption. Red arrowheads indicate clusters of electron dense aggregate material. All scale bars 100 nm.

The effect of pRLB‐540 on liposome integrity at low pH was further assessed by cryo‐electron microscopy (cryo‐EM) and tomography. Liposomes prepared in vitro were incubated with pRLB‐540 at 4 μM at pH 8.0 and 5.5 and plunge‐frozen after 20 and 60 s incubation. Liposomes were found to be ruptured after a 60 s incubation with pRLB‐540 at pH 5.5 (Figure 3c), but remained intact upon incubation with pRLB‐540‐noHis (Figure 3d). Small aggregates were visible for pRLB‐540‐noHis at pH 5.5, but to a much lesser extent than for pRLB‐540. Results from cryo‐EM and tomography are consistent with the liposome lysis assay and further demonstrate the pH‐dependent disruption of liposomes by pRLB‐540 (Figure 3c,d). To gain insight into early lysis events, and potentially into the mechanism of membrane lysis, cryo‐electron tomography (cryo‐ET) was performed on liposomes incubated with pRLB‐540 for 20 and 60 s at pH 5.5 (Figure 3e). Incubating pRLB‐540 with liposomes at pH 5.5 revealed the presence of aggregates after 20 s. Less aggregated material was observed at the 20 s time point than at the 60 s time point, in line with the kinetics observed in the dequenching assay. We also observed intermediate states of membrane lysis after 20 s where the liposome membrane was partly dissolved and lipids were released from the ordered structure of the lipid bilayer (Figure 3e; white arrowheads). pRLB‐540 may disrupt interactions between lipids upon insertion into the membrane and thus dissipate the ordered arrangement of the lipid bilayer. Based on these intermediate membrane disruption states observed by cryo‐EM, pRLB‐540 likely disrupts the lipid bilayer by micellization or the carpet‐mechanism (Fernandez et al., 2012; Sani & Separovic, 2016). After initial membrane disruption (20 s time point), aggregates of lipids and protein can be observed (60 s time point). From our observations during DLS experiments, we speculate that the proteins aggregate upon acidification if no hydrophobic environment such as a lipid membrane is present. Since we observed substantially less aggregated material at early time points of membrane lysis (Figure 3e; 20 s), we expect the exposed amphipathic helices to preferentially associate with lipid membranes.

AF2 predicted the hydrogen bond networks of pRLB‐540 with high similarity to the design model and high confidence (Figure 4a). The amphipathic helix of pRLB‐540 is more similar to membrane permeating peptides than the previously designed pRO‐2 I56V, which might contribute to its improved pH‐dependent lytic activity (Figure 4b). Helical wheel projections of the designs pRLB‐513, 515, and 518 show less spatial separation of hydrophobic and hydrophilic residues than pRLB‐540, which might explain differences in liposome lysis capacity (Figure S4).

**FIGURE 4:**
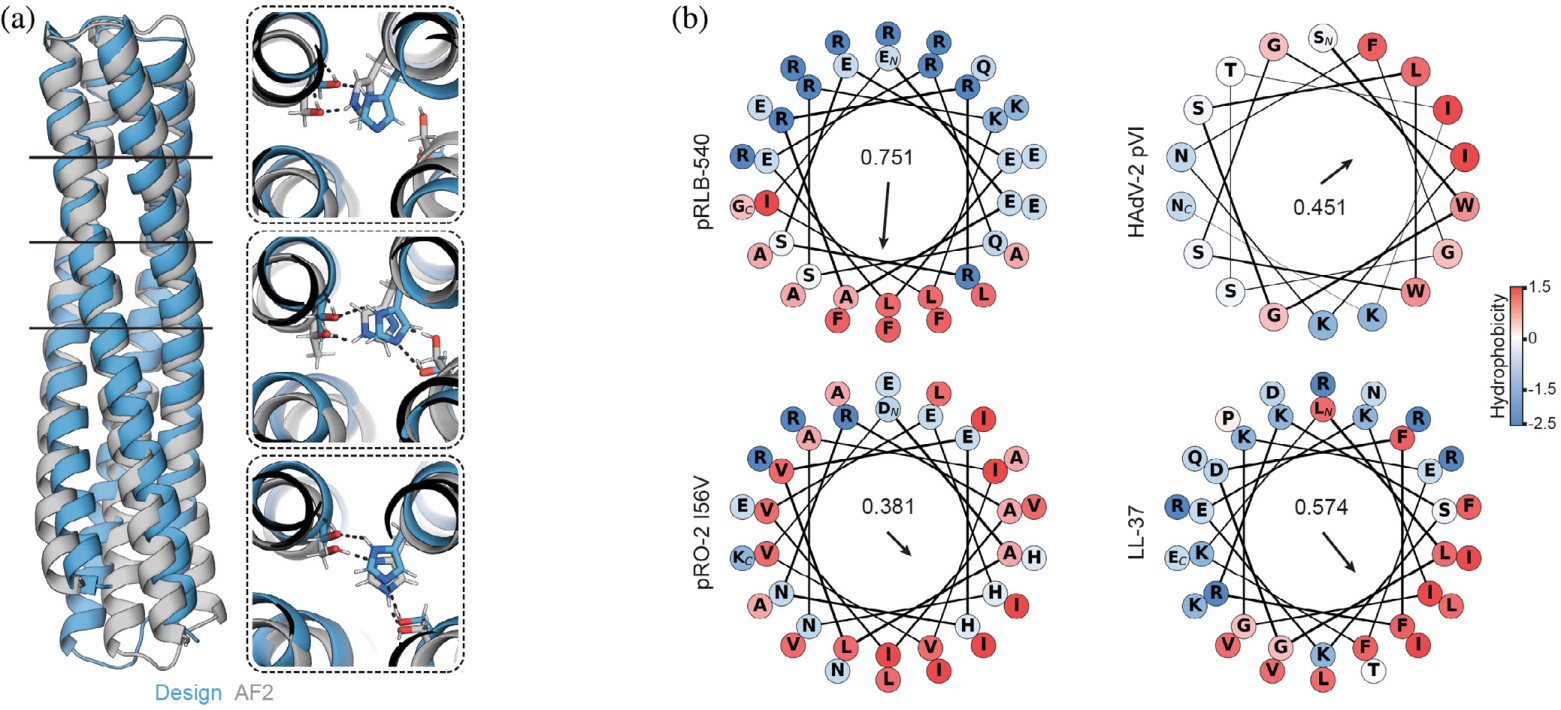
Structure analysis. (a) Comparison of pRLB‐540 design model to AF2 structure prediction. Boxes show details of the designed hydrogen bond networks. Black lines indicate the positions of these hydrogen bond networks in the bundle. (b) Comparison of amphipathic helices of pRLB‐540 and pH‐responsive bundles‐2 (pRO‐2) I56V to LL‐37 and pVI of human adenovirus 2 (HAdV‐2). The arrow represents the amphipathic moment of the helix, highlighting that design pRLB‐540 has a stronger moment than previously designed pRO‐2.

## 3 DISCUSSION

Endosomal escape remains a major barrier to intracellular delivery of biomolecules (Blanco et al., 2015). Many engineering efforts have used peptides, polymers, and lipid nanoparticles to address this challenge (Ahmad et al., 2021; Selby et al., 2017; Smith et al., 2019). Viral vectors allow robust intracellular delivery, but face pre‐existing immunity and in some cases difficult engineering (Li & Samulski, 2020). The de novo designed protein pRLB‐540 presented here lyses model membranes at pH 5.5, the pH of the late endosome before transition to the lysosome where cargo degradation may occur (Maxfield & Yamashiro, 1987). pRLB‐540 lyses artificial liposomes with efficiency similar to that of previously designed pRO‐2 I56V despite having fewer buried histidines, and has tighter pH control of lytic activity with no lysis at neutral pH. The correlation of liposome lysis with amphipathic moment supports the design concept used here and confirms the importance of amphipathic character of helices for membrane lysis. pRLB‐540 generated similar or higher levels of content leakage from artificial liposomes than antimicrobial peptides (Zhang et al., 2001), and approaches the lytic activity of the adenovirus protein VI, which was reported to lyse 80% of artificial liposomes at pH 6 (Blumenthal et al., 1986; Maier et al., 2010), while pRLB‐540 lysed 60% of artificial liposomes at pH 5.0. Incorporation of pRLB‐540, the most active design, on protein based nanocages, which is more straightforward than with our previous trimeric designs because it is monomeric and quite small, could help address the challenge of endosomal escape, a current bottleneck in cytosolic delivery by de novo designed nanocages.

## 4 MATERIALS AND METHODS

### 4.1 Computational methods

Backbones were generated by systematically varying the parameters of the Crick coiled coil equations (Bhardwaj et al., 2016; Crick, 1953). The helical radius was fixed to retain ideal ɑ‐helical geometry, helix length was fixed to 48 residues, and remaining parameters were sampled from native distributions (Grigoryan & Degrado, 2011). Hydrogen bond networks were then identified in the backbone library by using HBNet (Boyken et al., 2016; Maguire et al., 2018) in Rosetta as described previously (Boyken et al., 2019). Histidines were excluded from the C‐terminal helix and hydrogen bond networks were required to span at least three helices. Designs were filtered to contain two to three hydrogen bond networks. The helices were looped and the remaining sequence was designed using Rosetta design calculations (Alford et al., 2017; Bhardwaj et al., 2016; Dang et al., 2017; Fleishman et al., 2011; Hosseinzadeh et al., 2017; Khatib et al., 2011; Maguire et al., 2021; Tyka et al., 2011) or ProteinMPNN (Dauparas et al., 2022) while keeping residues participating in hydrogen bond networks fixed. Helices were designed to be amphipathic and contain phenylalanines for membrane interaction (Hosseinzadeh et al., 2017).

All Rosetta design scripts are available from https://github.com/ngoldb/pRLB.

### 4.2 Protein expression and purification

Designed protein sequences were ordered as synthetic genes in plasmids from Genscript Inc. (Piscataway, NJ, USA) or Integrated DNA Technologies Inc. (Coralville, IA, USA). Competent *E. coli* expression strain BL21(DE3) (NEB) were transformed with the plasmids and single colonies were picked for inoculation of starter cultures. Starter cultures were grown in lysogeny broth media containing 50 μg/mL kanamycin with shaking at 225 rpm for 8 h at 37°C. Five hundred milliliters of almost terrific broth media (12 g/L peptone and 24 g/L yeast extract, supplemented with trace metal mix, 50 × 5052, 20 mM MgSO_4_, and 10× phosphate buffer) were inoculated with 10 mL of starter culture. Proteins were expressed overnight by autoinduction (Studier, 2005) under antibiotic selection with shaking at 225 rpm at 30°C.

Cells were harvested by centrifugation at 4000*g* for 15 min at 12°C and lysed by sonication (4 min total, 10 s on–10 s off cycles, 80% amplitude) in lysis buffer (20 mM sodium phosphate buffer pH 7.4, 300 mM NaCl, 40 mM imidazole, 2 mM phenylmethylsulfonyl fluoride). Protein in the soluble fraction was purified by immobilized metal affinity chromatography. Lysates were cleared by centrifugation and the supernatant was incubated with 2 mL nickel‐nitrilotriacetic acid (Ni‐NTA) beads (Qiagen) for 20 min. Beads were washed with five column volumes of wash buffer (20 mM sodium phosphate buffer pH 7.4, 300 mM NaCl, 40 mM imidazole), followed by five column volumes of high salt wash buffer (20 mM sodium phosphate buffer, pH 7.4, 1M NaCl, 40 mM imidazole), and five column volumes wash buffer. Proteins were eluted in 4 mL elution buffer (20 mM sodium phosphate buffer pH 7.4, 300 mM NaCl, 250 mM imidazole) and ethylenediaminetetraacetic acid was added to a final concentration of 5 mM directly after elution. Eluted proteins were further purified by SEC on a Superdex 200 Increase 10/300 GL column (Cytiva) using 25 mM Tris–HCl pH 8.0, 150 mM NaCl. Peak fractions were verified by sodium dodecyl sulfate–polyacrylamide gel electrophoresis and SEC‐MALS and adjusted to a concentration of 5–10 mg/mL. Protein concentrations were determined by Qubit Protein BR assay (ThermoFisher). To identify the molecular mass of each protein, intact mass spectra were obtained via reverse‐phase liquid chromatography–mass spectrometry on an Agilent G6230B TOF on an AdvanceBio RP‐Desalting column, and subsequently deconvoluted by way of Bioconfirm using a total entropy algorithm. Protein samples were flash frozen in liquid nitrogen and stored at −80°C or stored at 4°C for immediate use.

### 4.3 SEC coupled to multi‐angle light scattering

Protein samples were diluted to 1–2 mg/mL and sterile filtered. SEC‐MALS was done on an Agilent 1200 high‐performance liquid chromatography coupled to a Heleos DAWN light scattering detector, Optilab rEX refractive index detector, and Nanostar DLS (Wyatt) using a Superdex 75 Increase 10/300 column (Cytiva) and 25 mM Tris–HCl pH 8.0, 150 mM NaCl. Data were analyzed using Astra software.

### 4.4 Circular dichroism

CD spectra were collected on a JASCO J‐1500 CD spectrometer. Protein samples were dialyzed into 25 mM NaCl, 15 mM sodium phosphate buffer of the respective pH (pH was adjusted using *o*‐phosphoric acid) at room temperature. Protein was diluted to a final concentration of 0.2 mg/mL and CD spectra were collected from 185 to 260 nm in a 1 mm pathlength cuvette. For temperature melts, protein samples were heated from 25 to 95°C at a rate of 1°C/s and spectra ranging from 185 to 260 nm were collected every 10°C. Every spectrum was baseline corrected using the respective buffer.

### 4.5 Liposome preparation

Liposomes with self‐quenching concentrations of SRB were prepared as previously published (Gui & Lee, 2018). In brief, 1,2‐dioleoyl‐sn‐glycero‐3‐phosphatidylcholine lipids at a concentration of 25 mg/mL (Avanti Polar Lipids) were diluted in chloroform and dried under vacuum in the dark overnight. The dried lipids were resuspended in Tris buffer (25 mM Tris–HCl pH 8.0, 150 mM NaCl, 2% glycerol) containing 25 mM SRB fluorophore (Sigma) to a final lipid concentration of 7.6 μM. The emulsion was subjected to 10 freeze–thaw cycles. The liposomes were passed 10 times through a 100 nm pore extruder (T&T Scientific Corporation). Intact liposomes were separated from free SRB using a PD‐10 desalting column following the manufacturer's instructions. Liposome preparations were validated to be monodisperse by DLS. Liposome diameters ranged from 80 to 120 nm with low polydispersity. The liposomes were stored at 4°C in the dark and used within 3 days.

### 4.6 Liposome disruption assay

Liposome disruption was assessed by fluorescence dequenching as previously described (Gui & Lee, 2018). Measurements were obtained on a FluoroMax 4 fluorometer (Horiba Instruments) using a quartz cuvette of 1 mm pathlength and excitation/emission wavelengths of 565/585 and 1 nm slit widths. Liposomes containing 25 mM SRB (self‐quenching concentration) were incubated with proteins (in 25 mM Tris–HCL pH 8.0, 150 mM NaCl, 2% glycerol) at a final concentration of 4 μM at pH 8 until the fluorescence signal stabilized. The solution was rapidly acidified to the respective target pH using acidification buffer (10 mM 4‐(2‐hydroxyethyl)‐1‐piperazineethanesulfonic acid, 150 mM NaCl, 50 mM citric acid, 0.02% NaN_3_, pH 3.0). The amount of acidification buffer to reach the respective target pH was previously determined by titration of the liposome storage buffer. Protein concentrations refer to concentration after acidification. Fluorescence signal was recorded until it remained stable and liposomes were fully lysed by addition of Triton X‐100 (Sigma) to a final concentration of 0.1%. Liposome disruption was measured by increase in fluorescence normalized to full lysis by Triton X‐100 and corrected to baseline:

$$\text{liposome disruption}=\frac{F_{t}-F_{0}}{F_{\max}-F_{0}},$$

where *F*_0_ refers to the average fluorescence intensity before acidification and *F*_max_ is the average fluorescence observed after addition of Triton X‐100.

### 4.7 Cryo‐EM and tomography

Samples pRLB‐540 and pRLB‐540‐noHis were prepared for cryo‐EM and cryo‐ET by plunge freezing by application to 200 or 300 mesh Quantifoil R 1.2/1.3 grids (EMS) and plunge frozen on a Vitrobot Mark IV (Thermofisher). Vitrobot chamber was maintained at a temperature of 4°C, 100% humidity, and a blot force of 0 and blot times between 4 and 5 s were used. The peptides were mixed with liposomes at pH 8.0, and the mixture was either maintained at pH 8.0 during plunge freezing or brought to pH 5.5 for 20 or 60 s at 4°C prior to plunge freezing. Conventional cryo‐EM was performed on a 120 kV Tecnai T12 TEM (FEI). Images were collected using the software Leginon (Suloway et al., 2005) at a nominal magnification of 67,000× corresponding to a pixel size of 1.60 Å/pixel at a defocus between 2 and 4 μm and a total electron dose 30 e−/Å (Wiethoff et al., 2005).

For cryo‐ET, tilt series were collected on a 300 kV Krios TEM (Thermofisher) equipped with a K3 direct electron detector (Gatan) and a post‐specimen BioQuantum GIF energy filter (Gatan). Tilt series were collected using Serial‐EM 4.0 (Mastronarde, 2005). Dose‐symmetric tilt series were collected between positive and negative 60°, with 3° increment at a nominal magnification of 64,000× corresponding to a pixel size of 1.4 Å/pixel. For each tilt, 0.3 s exposures were collected in movie‐mode with 0.05 s frames at a dose rate 15 electrons/pixel/second. Movies were motion‐corrected with MotionCor2 (Zheng et al., 2017) and tomograms were reconstructed using EMAN2 (nightly build) (Tang et al., 2007). IMOD v4.11 was used for visualization of images and tomograms (Kremer et al., 1996).

### 4.8 Crystal structure determination

All crystallization experiments were conducted using the sitting drop vapor diffusion method. Crystallization trials were set up in 200 nL drops using the 96‐well plate format at 18°C. Crystallization plates were set up using a Mosquito LCP from SPT Labtech, then imaged using UVEX microscopes and UVEX PS‐256 from JAN Scientific. Diffraction quality crystals formed in 0.2M sodium fluoride and 20% (w/v) PEG 3350.

Diffraction data were collected at the Advanced Light Source beamline on bl8.2.2. X‐ray intensities and data reduction were evaluated and integrated using XDS (Kabsch, 2010) and merged/scaled using Pointless/Aimless in the CCP4 program suite (Winn et al., 2011). Structure determination and refinement starting phases were obtained by molecular replacement using Phaser (McCoy et al., 2007) using the designed model for the structures. Following molecular replacement, the models were improved using phenix.autobuild (Adams et al., 2010); efforts were made to reduce model bias by setting rebuild‐in‐place to false, and using simulated annealing and prime‐and‐switch phasing. Structures were refined in Phenix (Adams et al., 2010). Model building was performed using COOT (Emsley & Cowtan, 2004). The final model was evaluated using MolProbity (Williams et al., 2018). Data collection and refinement statistics are recorded in Table S2. Data deposition, atomic coordinates, and structure factors reported in this paper have been deposited in the Protein Data Bank, http://www.rcsb.org/ with accession code 8GL3.

## CONFLICT OF INTEREST STATEMENT

Nicolas Goldbach, Issa Benna, Basile I. M. Wicky, and David Baker are inventors on a provisional patent application (63/499,405) submitted by the University of Washington for the design, composition, and function of the proteins created in this study.

## Supporting information


**Data S1.** Supporting Information.

## Data Availability

All data are available in the main text or as Supplementary material. The crystallographic dataset of pRLB‐519 has been deposited in the Protein Data Bank (PDB), with accession code 8GL3. Representative tomograms of liposomes incubated with pRLB‐540 have been deposited in the Electron Microscopy Data Bank (EMDB), https://www.ebi.ac.uk/emdb/, with accession codes EMD‐40185 (pH 8.0), EMD‐40186 (pH 5.5, 20 s), and EMD‐40187 (pH 5.5, 60 s).
