# Supplementary material for: De novo design of monomeric helical bundles for pH‐controlled membrane lysis
Source: Protein Sci. 2023 Oct 16;32(11):e4769. doi: 10.1002/pro.4769 (PMC10578055; doi:10.1002/pro.4769)
Supplement: Supplementary file 1 [file PRO-32-e4769-s001.docx]

# Supplementary material **De novo design of monomeric helical bundles for pH-controlled membrane lysis**

Nicolas Goldbach^1,2^**^‡^**, Issa Benna^1,3^**^‡^**, Basile I. M. Wicky^1,4^, Jacob T. Croft^5^, Lauren Carter^1,4^, Asim K. Bera^1,4^, Hannah Nguyen^1,4^, Alex Kang^1,4^, Banumathi Sankaran^6^, Erin C. Yang^1,4,7^, Kelly K. Lee^5,7,8^, David Baker*^1,4,9^

**^‡^** Equal contribution

* To whom correspondence should be addressed

**Table S1: Amino acid sequences of designed proteins**

| **Design name** | **Amino acid sequence** |
| --- | --- |
| pRLB-506 | MGSHHHHHHSGENLYFQGSEEEKEILEEIREILRKTEEIAEETERVAREAERVAREAQRAAELLERIAKNPNEEERRQIEEELEKAIEQLKRVIKKAQEVVRKNQRAVEENEEAARLAEELARRSPDEELQKAVRELKESVERLRESVKELERALREAEELLRELEEIAKGGSGSGENLYFQGGSGSLEEMARVAQEVVEALRKFVEIARRHVELLRRHREIAERFEELAKK |
| pRLB-507 | MGSHHHHHHSGENLYFQGSEEEKKQLERIQEILRQTEEIARETREIARKAREVAEEVERAAKLLEEIAKNPDPEEERKIQEEVERIAEELKRILEEVERAIRKNKEAVERNKEAVRIAERLAKRNKDEELRRAAKELKESVKRLEESVKEAEEAARRVQKEAERVREELRGGSGSGENLYFQGGSGSLEELARLAREAVRLVRELFELLREHIELLERHRELAERFREIAKK |
| pRLB-508 | MGSHHHHHHSGENLYFQGSEEEKEKAEEIEEIARETERLLEETERLVKKLEEVAEEVEKAARLAEELAKNKDEELEKEAKREAERVLELAKEVLRELEELVRKNKEAVEKNKRAARLAEELAKRSPDEEVRRAAKRARESVKRLEESVKELERLLEEAKRAAEELEELARGGSGSGENLYFQGGSGSLEEFAEVARQVLRAFRELREAAQRHIELMRRHLELIKELVRLLKK |
| pRLB-509 | MGSHHHHHHSGENLYFQGSEEEKEIAEEIERILRETEQVARETERIAEEAERVAELLERVARLLERLKKNPDEEERKKVEKEAREALERMKELLEEIRRIVELNKEVVRLNQEAAKKAREAAKKSGDEELRRLAEELQRSVERLEKSVREAEKAAREAERLARELEEEVRRGGSGSGENLYFQGGSGSLEELARLFKEAVELARRAVELLRRHVELLREHQEIARRFLELLRG |
| pRLB-510 | MGSHHHHHHSGENLYFQGSEEEKRELREVEKILEETREVAEKTEEIARELEKAARLIEEVARLVEEIAKNPDEEEEKRIRERAEEALREAERVLRELEEVVRENQELIRRNQEAARLAEEIARKSNDEELQEAARRLRESVEELEKSIKEVERAVRKAQKALKELEEEVRKGGSGSGENLYFQGGSGSLEELAEAFKRAAEILREVRELLQRHLELLRRHERLAEEFRRIAKK |
| pRLB-511 | MGSHHHHHHSGENLYFQGTEEERRALEEIREIAERTERIAEETERVARKLEEIAEELERVARLLERVAKNKNEEERKKAEQLLREALEEARKAAEELQEVVELNKEAVRQNQEAVRLAKELAKLNNDEEVERAAREIEESVKELERSVRELERILRELREALRRAEEAARRGGSGSGENLYFQGGSGSLEELARLAREVVRLAREAVEAFRRHVELLRRHVELVREFERLFKG |
| pRLB-512 | MGSHHHHHHSGENLYFQGSEEEQRQAEEIRKILEETKEAAERTREVAEEAERIAKEVEKAAKLLEEVAKNRDPEERRKAEEEAERVARLAEEILRKLEELVEENKELVKKNREAAELAERIAKRKNDEELRRAAERLRRSVEELERSVRELERLLREAQEVARELRELLKRGGSGSGENLYFQGGSGSPEELARLAKEAARMLKELVEAFRRHVELLEEHQKLAEEFERLVKG |
| pRLB-513 | MGSHHHHHHSGENLYFQGSEEEEEQLEKIRKILEETEELAEETERVAREAEEVVKEAQEAAELVEEIAKNPDEEERKRVREEVERILREAERLLRELERLARENKKAVERNQEAVRLAKELAKRKGDEELRRAAKRLEESVRELERSVRELERAIKRAQELLREAQEEVRKGGSGSGENLYFQGGSGSLEELAKLLQRLVELLRQLVELFKRHVELLRRHRELAQEFEEIAKK |
| pRLB-514 | MGSHHHHHHSGENLYFQGTEEEIRRLAEEHERLVREHERLLEEAERVAREAREVAEEAERILKSSDPEEEVRRRAEEAARRLEELARQLKRLAEELQESIRRLRESLKRAKEALKKNPSEEAKEAERLLEETERALEETERVLRKLKEVAERLEEAAELYKRARGGSGSGENLYFQGGSGSEEQERARKEFEELAKEFRELVEEFERVVELNRRLVEVNREVVELMKRG |
| pRLB-515 | MGSHHHHHHSGENLYFQGSEELREEAKRLAEEAERLLREHERILREVREVLEKHEKALEEARKALERAKRGEIPEEELKRVLEKLQRVAKETREALRRLQESLEKAKEALRLLKRALEIAKKLGLSEEEKKLAERAERVAREAEKVLRETERLAREAERILKETEELLREAEELLRGGSGSGENLYFQGGSGSEEEREEVLRKLEEALRRNQEAVKRLQEILRANEEVIKELQEVLKEFKRFMG |
| pRLB-516 | MGSHHHHHHSGENLYFQGSEEVKRELEEVAEELERVAKEHQELIEEHERLVKEAERIAEEAERRLAKPLPDEEREELARRLEELARRLEESVRRLQESLRKLQEALRKAKEALEKAKRQNPSEEQKRLLERAEEILREVERVARETEKLLEETARVARRAAELARKGGSGSGENLYFQGGSGSEEIRKVLRRLRELLRENEKFLQENRRLVEMVKEFRRIVERFRKG |
| pRLB-517 | MGSHHHHHHSGENLYFQGSEEERELREAAQRHEEALRRHEEILREAEEILEEVEEALKRAEELLKKGNLEEAARLMQEALKRAQEALRKVRESLEEIERSIRELEELLKRNRDSDEEKRRVLEEAERVLKETKRILEETRRLAEEAERLARRAEELARRIEERAKGGSGSGENLYFQGGSGSEEEKEKLRREAKEAIERFREAVEEFREAAEENRRAIRKNEEAIRLLKKA |
| pRLB-518 | MGSHHHHHHSGENLYFQGSEELREEAKRLAEEAQKIAEEHREVLERVEEVLKEHEEALKKAEEMLERLKRGEADDEEARRALEELLRVAEKTERALQELRESLQRLKRALELLRRALKLLKKLGLSEEERKVAEEIEKLAKEAQELAERTEELAKEAQEVLRETQRLLREARELLRNGGSGSGENLYFQGGSGSEEKERAARELEEALRRNKELVRRLQEALRQNKEALKRFEQAFREFQRLLG |
| pRLB-519 | MGSHHHHHHSGENLYFQGSEEAREVAEEILRLLREHEELLREHERLLKEARELAERLEELARRLEELARRGEKDEEAVRQVEEAAREAERVARELEKSARRLQESIRELRRLLKELRELLRELKKKASEEERKIAEELERIAEEAQRILEETERILRETVRIAQEAVRLLQEARRRAKKGGSGSGENLYFQGGSGSEEIEKLAREIKRAVEELQKALEENERAIRLNKEAARKFEEAVEKFRK |
| pRLB-521 | MGSHHHHHHSGENLYFQGTKEEAERIAKEAREILEEAEEILREAEEILRETEEALEETERALERLKKLPPEERQRLAERILEMNQRLAEKNREAVEKIREAVEKLKEAVEKLKEAVKKIKNSNPSEEEKKLAEEAERLARELEELAKRLEEAARKHKELIEEHLRLVRELVEALRGGSGSGENLYFQGGSGSEEEQERLRRELKESAKRLRESIREIERLAEEFRQAAEKFRKAAKKLG |
| pRLB-522 | MGSHHHHHHSGENLYFQGTREEVREILRKAREALERAEELLRRMEEILRETEEVLEETERALERAKTQPPEERERLAQRILELNQRAAEKNRRLVEELREAVEELKRIAEELKRAVELLKKSNPSEEEKELAREIEEVAREIERVAKELERAAREHKELIEEHLRLVREFVEAVKKGGSGSGENLYFQGGSGSEEQERLAEEIKESAKRLRESAERLRRIAEEFRRIAEEFRRIAKRFG |
| pRLB-523 | MGSHHHHHHSGENLYFQGTREEVREILEKAERILRKAEEILKEAEEILKRTEEVLKRTQEAAERLKKLPPEEARRLAERILRQNEEAARQNRELVRKLQEMVRELEQAVRELKEAVERLKKSNPSEEEKRLAEEIERVAREIERAARELRKAAEEHKRLIEKHLKLVREIVELLKKGGSGSGENLYFQGGSGSEEVRRALERLQESAEELRESARKLRELAEEFRKIAEEFRRIARKFG |
| pRLB-524 | MGSHHHHHHSGENLYFQGTEEEARELAEEAERILKKAEEILEEAERILEETERVLEETERALERLKTMPPEERRRLAEEILELNERLAQKNRELVEKLRKAVEELKEAVRRLKEAAKRLKNSNPSEEEKKLAEELEEAAKEIEEVAKRIEEAAREHKRLIEEHLKLVRELVEALKKGGSGSGENLYFQGGSGSEELERLARELKESAKRLRESARRLRELAEEFRRIAEKFRQIARRFG |
| pRLB-525 | MGSHHHHHHSGENLYFQGSERERARRLLEEAQRLAQEARELLREAQEILQEHREKLQEHRELLEELRKKNRIDPEEAEKSRRLAEESRQLARRLQELVRRAQELARRLRELVEELKKIPELSEEAKRLLEEAERLAEEIERLLRRIEELLRELERVSKELRELSREAEELARNGGSGSGENLYFQGGSGSEERARRILKRLQELSERLQRLSEEAQRLARRAEELLREFQEFAERARKLA |
| pRLB-526 | MGSHHHHHHSGENLYFQGSEEEEEARRILEELERLARELEELARRAEEILRKSERIIRRSEEAAERIRRGEIPPEERKRLAEEAARLVREAQRLVEEIAKALERAQEVVERARRLAELIKKKSRDEEVRRVAEELERVAQELQRLARRAAELARRHAKLLEEHARLLERIRKGGSGSGENLYFQGGSGSEEEARRRLQESAERLKRSLEEAKRALEEFKKLLERFRRLAERLRRLG |
| pRLB-527 | MGSHHHHHHSGENLYFQGMKALEKLKKVKEEIKKVEEEVKKAEEEVEKSKEKAKESKEEAEKLGKGAIEGKFDAEGGKKLAEKNEKLAEENEKMAEELEEAAEKAIKAAEEAEEAAKELKEEAKDEEEKEKAEELKEAAKKLKEAAEELKKAAEEHKEKAEEHKEKAKKFKEVVEKYERGGSGSGENLYFQGGSGSGEEVAKAAKELEESAEELEESAEKLEEAIKKFKEAVKEFDEAIEKLE |
| pRLB-528 | MGSHHHHHHSGENLYFQGMEAKEKIKKAKEELKKVEEEAKKAEEGIKKSEEKAKESKEKAEELGEGAKKGKFDVEGGEALAEENEKLAKENEELAEEMIKAAKEAEEAAKELEEAAKEIEEKAEDEEEKEKAKELKEAAKKLKEAAEELKKVAEEHKKVAEEHEKVAEKFKEVIEKYKKGGSGSGENLYFQGGSGSGEEVYKAAEELVESAEKFEESAKKMEEAIKKFREAVKEVDEAIKKFE |
| pRLB-529 | MGSHHHHHHSGENLYFQGMAAREEVARVKREVEEVRAEAERIEAEVRESEERARESRERARELGRGAREGEVDVEGGRELARRNEELARRNEEAAERAKEAAERAEEAAREAERAAEKLKEEAKREEEAEKAEKLAAAARALREAARAFREAAERHEAVARRHEEAARRFREVLERYERGGSGSGENLYFQGGSGSGEEVARAAEELERSADELEASADRVRAAIEAFRAAVEAFDRAVEELE |
| pRLB-530 | MGSHHHHHHSGENLYFQGMKAKEELKKVLEEVEKVEEKAEEIKEKVEESREKAEKSKEEAEKLGEGAKKGEFDAEGGKKLAEENLELAKENEEMAKEAIEAAEELEEAAKKAKEAAEKLEKEAAKEEEAEKAKELKEAAEELKEAAKEFKEKAEEHRKKAEEHKKVAEEFKKVVEKYEEGGSGSGENLYFQGGSGSGEEVYEAAEKLVESAEKLEESAEEMEEAIEELKEAIEKFKEAVKKYE |
| pRLB-531 | MGSHHHHHHSGENLYFQGARLAELARVLREAAEEGKKMVALGKEAEELLDEHLELLEEHEKLLADRGGDEEKRGEELAKSAEKLVESAEKMEEYAKEMKKGAEELEAAAARARELLEDRPAGRAAAAALRRALEAVKEAKEKFEKAEELFKKSGELMRESGELAKGGSGSGENLYFQGGSGSFERAEELSKKAKELSEKAAELMKEAAEKMKAAAEELEAAGRRLAEE |
| pRLB-532 | MGSHHHHHHSGENLYFQGMEKLEEIKKLMEELKELVDELEELVEEYEEEIERYEEKIEEYKKDIEKYIEGEVEWEEGKERSKKYIEESERYIKESREYIKELEEKIKKIKELIEKLKEMLGDDEDPKVKELLELLKKLEEKLKELEELLKEHEEEIEKHEEVLKKHEEDLKYGGSGSGENLYFQGGSGSEEEKEKLKKSKEELEKSKERLRESAEELEEFAEKLKELADKIEELAKEL |
| pRLB-537 | MGSHHHHHHSGENLYFQGSEEEEKLRRAAEEAERIAEEAERILEETKRVLEETRRILEETERALKKGAPPEEVQRLARENRRAAEENRRLAEENRELVKKLERLAEKLKKLAQPSEEARKMKEIVEELEKAAQEHRRIVEEHQKLAEEHQRLAEEVEELAKGGSGSGENLYFQGGSGSEEARKRLKEAAERLQESAQRLQESIRKLEESIRRLEQLARELKEALKRLKG |
| pRLB-538 | MGSHHHHHHSGENLYFQGSEEEKKLREALEEAERALKEAEEILEETERLLKETEEILRETEEALKKGVPPEEVRRLARENREAAERNEEVARQNKRMVEELERIAEELKRLSQPTEEAKEAQRIAEEIERIAREHKRLVEEHERLVREHREIAERVEEAAKGGSGSGENLYFQGGSGSEEAKEELEELRRRLEESAKKLRESIRKLEESARRFREAAERFREVAERLRG |
| pRLB-539 | MGSHHHHHHSGENLYFQGDREELEELVRRAEELAEELRRIAEEHERVSREFERVSRKVERAARSPIPKEERKKVAEELERALEKLKRVLRESQRALEMAKEALRLLKEALKLAREIKKKADSEEERRAIEALERAAELLEEAERLLEEAERLLRESEELLKVTEELLELLKGGSGSGENLYFQGGSGSPEEEKRLLEEHREALERHRRATEIFKEAVELFEEALRVAREALKG |
| pRLB-540 | MGSHHHHHHSGENLYFQGSEEERARELLKRARELLEEAERLLREHEELLEEHRRALEEHQELLERAKKLGEEELKEAARRSEQVLRITEEVARRSEEIARRVEKAAREIREMAEELRKNVPDSEEARKIAQELREIAERLQEVAKRFREVVQRAREILERIARILREIIELLRGGSGSGENLYFQGGSGSEEALRRARELSEEARRRSEELERQSRKLIREFEQLAREFRRAARKFG |

**Table S2: Crystallographic data collection and refinement**

|  | **pRLB-519 (8GL3)** |
| --- | --- |
| **Data Collection** |  |
| Space group | I 1 2 1 |
| *Cell dimensions* |  |
| *a*, *b*, *c* (Å) | 32.12, 44.70, 182.57 |
| *α*, *β*, *γ* (°) | 90, 90.43, 90 |
| Resolution (Å) | 45.64 - 2.3 (2.53 - 2.30) |
| *R_merge_* | 0.082 (0.144) |
| *R*_pim_ | 0.052 (0.091) |
| *I/σ*(*I*) | 10.50 (7.39) |
| *CC* _1/2_ | 0.998 (0.976) |
| Completeness (%) | 98.36 (98.13) |
| Redundancy | 3.3 (3.4) |
| **Refinement** |  |
| Resolution (Å) | 45.64 - 2.3 (2.53 - 2.30) |
| No. reflections | 11501 (2812) |
| *R*_work_ / *R*_free_ | 0.2388 (0.2370)/ 0.2832 (0.3090) |
| *No. atoms* |  |
| Protein | 1600 |
| Water | 31 |
| Ramachandran Favored/allowed/Outlier (%) | 100.00/ 0.00/ 0.00 |
| *R.m.s. deviations* |  |
| Bond lengths (Å) | 0.002 |
| Bond angles (°) | 0.350 |
| *B_factors_ (Å^2^)* |  |
| Protein | 46.29 |
| Water | 33.11 |

**Figure S1 | Experimental characterization of designed proteins.** SEC chromatogram (**A**), CD spectra (**B**) and DLS (**C**) of designed proteins.

**Figure S2 | CD temperature melt** (**A**) Protein pRLB-540 (**B**) Protein pRLB-539 at pH 7 and 5

**Figure S3 | Liposome lysis of pRLB-540 and variants compared to pRO-2 I56V** (**A**) Fluorescence dequenching of SRB liposomes upon acidification to pH 5. No protein present. Blue arrowhead indicates addition of Triton-X100. Acidification to pH 5 at 0 seconds. (**B**) Comparison of liposome lysis of pRLB-540 to pRO-2 I56V at pH 5. (**C**) Comparison of liposome lysis of pRLB-540 to pRO-2 I56V at pH 7.3, 5.9, 5.5, and 5. Red arrowheads indicate acidification to respective pH.

**Figure S4 | Comparison of amphipathic helices** Amphipathic helices (36 C-terminal residues) of pRLB-540 (**A**), pRLB-518 (**B**), pRLB-515 (**C**), pRLB-513 (**D**). Arrow and numbers show direction and magnitude of the amphipathic moment. Residues are colored by hydrophobicity.
